# Adaptation and validation of the memorial anxiety scale for prostate cancer (MAX-PC) in a sample of French men

**DOI:** 10.1186/s41687-019-0150-1

**Published:** 2019-09-06

**Authors:** Rajae Touzani, Julien Mancini, Jaïs Troïan, Anne-Déborah Bouhnik, Olivier Cussenot, Gwenaelle Gravis, Patricia Marino

**Affiliations:** 1 Institut Paoli-Calmettes, SESSTIM U1252, Marseille, France; 2 Aix Marseille Univ., INSERM, IRD, SESSTIM, Sciences Economiques & Sociales de la Santé & Traitement de l’Information Médicale, Marseille, France; 3 Aix Marseille Univ., APHM, INSERM, IRD, SESSTIM, Sciences Economiques & Sociales de la Santé & Traitement de l’Information Médicale, Hop Timone, BioSTIC, Biostatistique et Technologies de l’Information et de la Communication, Marseille, France; 4 Aix-Marseille Univ., Laboratoire de Psychologie Sociale (LPSEA849), Aix-en-Provence, France; 5 APHP, Hôpitaux Universitaires Est Parisien, Service Urologie, Sorbonne Université, Paris, France; 6 Medical Oncology, Institut Paoli Calmettes, Centre de Recherche en Cancérologie de Marseille, Aix Marseille Université, Marseille, France

**Keywords:** MAX-PC, Prostate cancer, Validation, French men

## Abstract

**Introduction:**

The Memorial Anxiety Scale for Prostate Cancer (MAX-PC, 18 items) was developed to assess anxiety in prostate cancer patients. In the absence of a French version of this scale, we adapted the original English scale and evaluated its psychometric properties in a sample of French men diagnosed with prostate cancer in the previous 12 months.

**Methods:**

The MAX-PC was translated from English to French and distributed online by two non-profit organizations (*Seintinelles* and *ANAMACaP*). The French questionnaire, which also included the Hospital Anxiety and Depression Scale (HADS) and a measure of health-related quality of life (SF12), was intended for adults diagnosed with prostate cancer in the previous 12 months. Factor structure was assessed using exploratory factor analysis (EFA) on data from 56.2% of the sample (*n* = 104, *Seintinelles* subsample) and confirmed using confirmatory factor analysis (CFA) on data from the rest of the sample (*n* = 81, *ANAMACaP* subsample). The reliability of the scale was measured using Cronbach’s alpha coefficient. Construct validity was assessed by calculating the correlation of the MAX-PC total score and subscale scores with the HADS total score and subscale scores and with the SF12 total score and subscale scores.

**Results:**

Of the 185 respondents, 168 (90.8%) had complete data on all MAX-PC items. The average age of participants was 65.1 years (SD: 7.7). The three-factor structure defined in the original validation study was very similar in EFA and then confirmed by CFA. The MAX-PC showed good reliability, as Cronbach’s alpha coefficients for the scale and for its three subscales were 0.92, 0.90, 0.68, and 0.87, respectively. It also showed good construct validity. As expected, the MAX-PC total score was positively correlated with the HADS-Anxiety subscale score (r = 0.68, *p* < 0.001) and negatively correlated with the SF12-MCS subscale score (r = − 0.35, *p* < 0.001).

**Conclusion:**

The French version of the MAX-PC shows adequate psychometric properties among French men with prostate cancer. This scale may be used in future studies and in routine clinical care to help health care providers identify patients who need psychological support due to prostate-cancer related anxiety.

## Introduction

Prostate cancer is an important public health concern. It is the most common cancer among men and the 6th leading cause of cancer death worldwide [1]. In 2012, 307,000 deaths were recorded as a result of the disease [2]. France has the third highest incidence rate of prostate cancer among EU countries [3] (INCA 2017). Although the death rate has significantly decreased since the 1990s, prostate cancer remains the third leading cause of cancer death in French men [4].

The management of patients with prostate cancer varies according to the stage of the disease and can involve surgery, radiotherapy (with or without androgen deprivation therapy), brachytherapy, or active surveillance for localized prostate cancer. It can also vary according to patients’ preferences, as different therapeutic options are available that produce similar benefits based on purely clinical or biological parameters. However, the adverse effects of the different treatments (fatigue, nausea, pain, loss of social/physical and sexual capabilities) vary significantly depending on the mode of patient management [5]. They can also have a profound psychological impact on patients [6]. Thus, one study found that the risk of suicide was 4 times higher among men with prostate cancer than among healthy men [7]. In a context where prostate cancer management is becoming increasingly complex, patient reported outcome measures (PROMs) constitute a useful tool to assess therapeutic strategies and to improve quality of care and patients’ health-related quality of life [8].

Studies have shown that reception of diagnosis and changes in the mode of care increase anxiety in men with prostate cancer [9]. Many patients express feelings of stress, worry, and anxiety when they learn that they have prostate cancer or when they are asked to choose between different treatments [10], with anxiety rates ranging from 15.1% to 32.6% [11, 12]. However, prostate cancer-related anxiety is usually estimated using general anxiety scales, namely the Hospital Anxiety and Depression Scale (HADS) [13], the Brief Symptom Inventory (BSI) [14], or the Distress Thermometer (DT) [15]. Because these scales were not specifically designed for prostate cancer patients, they fail to take into account the specific context of prostate cancer. Indeed, the latter is characterized by the use of repeated prostate-specific antigen (PSA) tests, which are known to generate a unique form of anxiety [16].

In light of these challenges, Roth et al. [17] developed and validated a new PROM, the Memorial Anxiety Scale for Prostate Cancer (MAX-PC), to help accurately measure anxiety in patients with prostate cancer.

The aim of this study was to propose a French version of the MAX-PC and to evaluate its psychometric properties in a sample of French men diagnosed with prostate cancer in the previous 12 months.

## Methods

### Description of the MAX-PC

The original MAX-PC was developed by Roth et al. in the US in 2003 [17].

The MAX-PC consists of 18 items divided into three subscales. The first subscale is composed of 11 items measuring prostate cancer anxiety (PCA) and the second of three items measuring anxiety related to PSA testing (PSAA). The items of the first two subscales are scored on a 4-point Likert scale (0: Not at all; 1: Rarely; 2: Sometimes; and 3: Often). The third subscale measures anxiety related to fear of recurrence (FOR), and is composed of 4 items scored on a different 4-point Likert scale (0: Strongly disagree; 1: Disagree; 2: Agree; and 3: Strongly agree). The MAX-PC total score ranges from 0 to 54, while the PCA, PSAA, and FOR subscale scores range from 0 to 33, 0 to 9, and 0 to 12, respectively. Men who score above 27 on the MAX-PC are identified as clinically anxious [18].

### Translation

The MAX-PC was translated from English to French using the back-translation method [19]. Two French native speakers with a good level of scientific and medical English translated the scale separately. Both versions were compared and reconciled into a single version following a consensus meeting of experts (three researchers and one clinician specialized in prostate cancer care). This version was then back-translated by a native English translator to ensure translation equivalence. Lastly, the French translation and the back-translation were reviewed and compared to obtain a final French version of the scale.

The French version of the MAX-PC and the full questionnaire were tested face-to-face with 11 patients at the Regional Comprehensive Cancer Center of Marseille (Paoli-Calmettes Institute) to check for feasibility and acceptability. The 11 respondents answered all 18 items of the MAX-PC without any difficulty. The full questionnaire was completed in 15 min on average. Following this pilot study, a few minor textual changes were made to the final questionnaire (no changes were made to the MAX-PC).

### Participants

Eligible participants were adult men living in France who had been diagnosed with prostate cancer in the previous 12 months. The questionnaire was administered in collaboration with two non-profit organizations: Seintinelles [20] and ANAMACaP (Association NAtionale des MAlades du CAncer de le Prostate) [21]. Seintinelles is a collaborative research platform that aims to accelerate cancer research. The platform sets up researchers and 27,451 citizens (non-sick, sick but also those who have never been affected by cancer), including 5% of men. Seintinelles emails its newsletter with information about the studies for which the recruitment of participants is open. ANAMACaP is a French prostate cancer patient advocacy group including more than a thousand members (prostate cancer patients and their relatives). Our completely anonymous questionnaire was hosted on a secure webpage, and an email containing a hyperlink to the questionnaire was sent to members of both organizations between April 2016 and March 2018. This procedure allowed us to generate two subsamples, one for each organization. This study was approved by the National Commission for Information and Freedoms (Commission Nationale de l’Informatique et des Libertés, CNIL: 1955704) and by the Paoli-Calmettes Institute review board (COS: IPC 2016–009).

### Questionnaire

In addition to the MAX-PC, the online questionnaire included the HADS (Hospital Anxiety and Depression Scale), a scale designed to detect anxiety and depressive disorders (14 items rated from 0 to 3, including 7 items related to anxiety and 7 to depression) [13], and the SF12 (Short Form-12), a survey measuring health-related quality of life (12 items divided into two subscales: the PCS (Physical Component Score) and the MCS (Mental Component Score)) [22]. As our online questionnaire was strictly anonymous, the only collected socio-demographic variable was patient age.

### Statistical analyses

The factor structure of the MAX-PC was assessed using Exploratory Factor Analysis (EFA) on the first subsample (Seintinelles) and then confirmed using Confirmatory Factor Analysis (CFA) on the second subsample (ANAMACaP). Given that the items of the MAX-PC scale are ordered categorical variables, we generated a matrix of polychoric correlation to carry out a factor analysis [23, 24]. The robust weighted least squares estimator was used for both EFA and CFA [24, 25].

EFA using oblique rotation was performed on the first subsample to replicate the three-factor structure defined in the original validation study by Roth et al. [17]. Factor adequacy was assessed using Bartlett’s sphericity and the Kaiser-Meyer-Oklin (KMO) tests [25, 26].

CFA was then performed on the second subsample to confirm the structural validity of the MAX-PC scale. CFA model fit was assessed using the following goodness of fit indices: root mean square error of approximation (RMSEA, good fit if < 0.05) [27], comparative fit index (CFI, good fit if close to 1) [28] and Tucker-Lewis index (TLI, good fit if close to 1) [29, 30].

Scores were expressed as mean, standard deviation, median, and percentage of respondents who had the highest and lowest scores on the different scales. Ceiling and floor effects were considered present when more than 15% of participants had the highest possible score or the lowest possible score, respectively [31].

The reliability and construct validity of the MAX-PC were estimated on the entire sample. Reliability was measured as internal consistency [32] by calculating Cronbach’s alpha coefficient for the MAX-PC and its three subscales (PCA, FOR, and PSAA). A Cronbach’s alpha Coefficient ≥ 0.7 was considered satisfactory [33]. Convergent validity was assessed by calculating the correlation (Pearson coefficient) of the MAX-PC total score and subscale scores with the HADS-Anxiety subscale score, the HADS-Depression subscale score and the SF12-MCS subscale score. Discriminant validity was assessed by calculating the correlation of the MAX-PC total score and subscale scores with the SF12-PCS subscale score. Pearson correlation coefficients whose r ≥ 0.30 were considered relevant [34].

We hypothesized that the MAX-PC total score and subscale scores have to be strongly positively correlated with the HADS-Anxiety subscale score, the HADS-Depression subscale score [12, 35] and negatively correlated with the SF12-MCS subscale score [12, 36]. We also expected the MAX-PC total score and subscale scores to be weakly correlated with the SF12-PCS subscale score.

All statistical analyses were performed using STATA/SE 12.0 and R (version 3.6.0) softwares.

## Results

A total of 185 men completed the online questionnaire. Of these, 104 were recruited by *Seintinelles* and 81 were members of the *ANAMACaP*. The average age was 65.1 years (SD = 7.7) with no different between the two samples.

The highest percentage of missing data was 1.6% for items 6 and 11 (Table 1). All participants responded to item 2 (“Even though it’s a good idea, I found that getting a PSA test scared me”). Three items had a median score of zero (items 12, 13 and 14). The range observed for all items was 0 to 3.

**Table 1:** Descriptive statistics for each item on the MAX-PC (*N* = 185)

Item	Label	*N*	% missing	Median	Observed range
1	Any reference to prostate cancer brought up strong feelings in me.	184	0.5	2	0–3
2	Even though it’s a good idea, I found that getting a PSA test scared me.	185	0.0	1	0–3
3	Whenever I heard about a friend or public figure with prostate cancer, I got more anxious about my having prostate cancer.	183	1.1	1	0–3
4	When I thought about having a PSA test, I got more anxious about my having prostate cancer.	184	0.5	1	0–3
5	Other things kept making me think about prostate cancer.	183	1.1	1	0–3
6	I felt kind of numb when I thought about prostate cancer.	182	1.6	1	0–3
7	I thought about prostate cancer even though I didn’t mean to.	184	0.5	2	0–3
8	I had a lot of feelings about prostate cancer, but I didn’t want to deal with them.	184	0.5	1	0–3
9	I had more trouble falling asleep because I couldn’t get thoughts of prostate cancer out of my mind.	184	0.5	1	0–3
10	I was afraid that the results from my PSA test would show that my disease was getting worse.	183	1.1	2	0–3
11	Just hearing the words “prostate cancer” scared me.	182	1.6	1	0–3
12	I have been so anxious about my PSA test that I have thought about delaying it.	184	0.5	0	0–3
13	I have been so worried about my PSA test result that I have thought about asking my doctor to repeat it.	184	0.5	0	0–3
14	I have been so concerned about my PSA test result that I have thought about having the test repeated at another lab to make sure they were accurate.	184	0.5	0	0–3
15	Because cancer is unpredictable, I feel I cannot plan for the future.	184	0.5	1	0–3
16	My fear of having my cancer getting worse gets in the way of my enjoying life.	183	1.1	1	0–3
17	I am afraid of my cancer getting worse.	184	0.5	2	0–3
18	I am more nervous since I was diagnosed with prostate cancer	184	0.5	2	0–3

### Exploratory factor analysis

Bartlett’s test of sphericity was significant (χ^2^ = 5231.9, dl = 153, *p* < 0.001) and the KMO test for sampling adequacy indicated a very good fit for factor analysis (0.87). The factorial structure was similar to that of the original English version, except for a single item (2). Items 1 to 11 (factor 1) loaded higher on the PCA subscale than on the other two subscales (≥0.42). Items 15 to 18 (factor 2) all loaded higher on the FOR subscale, with each item loading > 0.53. Items 12, 13 and 14 (factor 3) all loaded higher on the PSAA subscale, with each item loading ≥0.40. In contrast, Item 2 loaded higher on the PSAA subscale (0.45 vs. 0.42 on the PCA subscale). EFA results are detailed in Table 2.

**Table 2:** Factor analysis of the MAX-PC with oblique rotation (*N* = 98)^a^

Item	Factor loading		
	PCA	FOR	PSAA
1	0.49		
2	0.42		0.45
3	0.54		
4	0.64		
5	0.73		
6	0.86		
7	0.77		
8	0.66		
9	0.58		
10	0.68		
11	0.64		
12			0.40
13			0.68
14			0.73
15		0.84	
16		0.96	
17		0.52	
18		0.55	
Eigenvalues	5.39	2.82	1.80

*PCA* Prostate cancer anxiety subscale, *FOR* Fear of recurrence subscale, *PSAA* PSA anxiety subscale

^a^ Analysis performed on the sample without missing data (listwise method, N = 98)

Only loadings ≥ 0.40 are presented

Primary factor loading is denoted by italic text

Original version of the MAX-PC: Items 1, 2, 3, 4, 5, 6, 7, 8, 9, 10, and 11 belong to the PCA subscale; items 12, 13, and 14 belong to the PSAA subscale; items 15, 16, 17, and 18 belong to the FOR subscale

### Confirmatory factor analysis

To confirm the structural validity of the MAX-PC, the factor structure obtained by EFA was tested and compared to the original structure using CFA on the second subsample removing all subjects with missing data (listwise method, ANAMACaP sample, *N* = 70). The CFA of the original structure had reasonable fit, with RMSEA = 0.079 (90% Confidence Interval: 0.052–0.104), CFI = 0.957, and TLI = 0.950. Following the study of the modification indices (MI), we added those whose MI value was greater than 10 and which seemed to have a theoretical sense. The correlated measurement errors selected was between items 2 and 4 (14.2) and items 13 and 14 (10.1). We added these two correlated measurement errors as “free parameters” which significantly improved the fit indices (nested model test, *p* < 0.001): RMSEA = 0.073 (90% Confidence Interval: 0.043–0.098), CFI = 0.964, and TLI = 0.958.

A second factor structure was also tested by CFA, in which item 2 was moved from the PCA subscale to the PSAA subscale according to the new structure suggested by the EFA. The fit indices of this model were: RMSEA = 0.082 (90% Confidence Interval: 0.056–0.106), CFI = 0.954, and TLI = 0.946. We added two highest correlated measurement errors as free parameters (MI = 20.2 between items 2 and 4, MI = 11.6 between items 13 and 14) which significantly improved the fit indices (*p* < 0.001): (RMSEA = 0.078 (90% Confidence Interval: 0.040–0.111), CFI = 0.960, and TLI = 0.955), but remaining lower than those of the original structure. Testing a model with item 2 cross-loaded in the two factors (PCA and PSAA) and taking into account the MI (items 2 and 4: 30.1), we observed a very little no significant variation in fit indices compared to the original structure (*p* = 0.531): RMSEA = 0.074 (90% Confidence Interval: 0.045–0.100), CFI = 0.963, and TLI = 0.956.

In light of this, the original structure was retained for the rest of the analyses.

### Reliability analysis

The different scores and their distributions are detailed in Table 3. A total of 168 men (90.8%) responded to all 18 MAX-PC items. The average MAX-PC total score was 22.7 (SD: 12.2), and 36.3% of sample participants had clinically significant anxiety (score > 27). No ceiling effects were observed. Only the PSAA subscale exhibited strong floor effects as 60.3% of respondents had the lowest score (item 12: 76.6%; item 13: 69.6%; and item 14: 80.4%), indicating a lack of anxiety related to PSA testing.

**Table 3:** Scores and distributions (N = 185)

	*N*	Mean	SD	Median	Observed score range	% Minimum score	% Maximum score	Possible score range
MAX-PC	168	22.7	12.2	23.0	0–47	1.8	0.6	0–54
PCA	171	15.7	8.6	16.0	0–32	2.3	1.2	0–33
PSAA	184	1.2	1.9	0.0	0–7	60.3	0.5	0–9
FOR	183	5.8	3.3	6.0	0–12	5.5	4.9	0–12
HADS	175	13.9	7.8	13.0	1–35	1.1	0.6	0–42
HADS-Anxiety	180	8.7	4.5	8.0	0–20	0.6	1.1	0–21
HADS-Depression	180	5.2	4.1	4.0	0–19	8.9	0.6	0–21
SF12	177	45.3	6.6	45.2	16.4–59.9	0.6	0.6	0–100
SF12-PCS	177	46.0	9.0	47.1	18.6–62.3	0.6	0.6	0–100
SF12-MCS	177	46.4	8.8	46.3	23.8–72.7	1.0	1.0	0–100

*MAX-PC* Memorial anxiety scale for prostate cancer, *PCA* Prostate cancer anxiety subscale, *PSAA* PSA anxiety subscale, *FOR* Fear of recurrence subscale, *HADS* Hospital Anxiety and Depression scale, *HADS-Anxiety* HADS anxiety subscale, *HADS-Depression* HADS Depression subscale, *SF12* Short Form-12, *SF12-PCS* SF12 physical component subscale, *SF12-MCS* SF12 mental component subscale

Cronbach’s alpha coefficient indicated adequate reliability for the MAX-PC (α = 0.92), the PCA subscale (α = 0.90), and the FOR subscale (α = 0.87). However, the internal consistency of the PSAA subscale was rather low (α = 0.68) (Table 4).

**Table 4:** Cronbach’s alpha coefficients for the French version of the MAX-PC and for other published versions

	French version^a^	Original version^b^	Italian version^c^	Dutch version^d^	Chinese version^e^
PCA	0.90	0.90	0.88	0.91	0.93
PSAA	0.68	0.54	0.61	0.64	0.82
FOR	0.87	0.85	0.78	0.85	0.85
MAX-PC	0.92	0.90	0.91	0.77	0.94

*PCA* Prostate cancer anxiety subscale**,** *PSAA* PSA anxiety subscale**,** *FOR* Fear of recurrence subscale**,** *MAX-PC* Memorial anxiety scale for prostate cancer

^a^ Cronbach’s alpha coefficients were computed on the entire sample without missing data (N = 168)

^b^(Roth AJ. 2003)

^c^(Alvisi MF 2017)

^d^(Van Den Bergh RC 2009)

^e^(Huang Q 2017)

### Construct validity analysis

Table 5 shows the correlation of the MAX-PC total score and subscale scores (PCA, FOR, and PSAA) with the HADS total score and subscale scores (HADS-Anxiety and HADS-Depression), with the SF12 total score and subscale scores (SF12-MCS and SF12-PCS), and with age. Only two relevant correlations (r ≥ 0.30) were found for the PSAA subscale score. The MAX-PC total score and the other two subscale scores (PCA and FOR) were significantly correlated with the HADS total score and subscale scores and with the SF12 total score and subscale scores. The correlation of the MAX-PC total score with the HADS total score, the HADS-Anxiety subscale score, and the HADS-Depression subscale score was r = 0.69 (*p* < 0.001), r = 0.68 (*p* < 0.001), and r = 0.58 (*p* < 0.001), respectively. The MAX-PC total score was negatively correlated with the SF12 total score (r = − 0.48; *p* < 0.001), the SF12-MCS subscale score (r = − 0.35; *p* < 0.001), and the SF12-PCS subscale score (r = − 0.32; *p* < 0.001). As expected, the MAX-PC total score and subscale scores demonstrated a weaker correlation with SF12-PCS compared to HADS-Anxiety.

**Table 5:** Criterion and construct validity of the French version of the MAX-PC (Pearson correlation coefficients, *N* = 185)

	MAX-PC	PCA	PSAA	FOR
HADS	0.69**	0.62**	0.30**	0.74**
HADS-Anxiety	0.68**	0.64**	0.30**	0.69**
HADS-Depression	0.58**	0.50**	0.25**	0.66**
SF12	−0.48**	−0.41**	−0.18*	−0.53**
SF12-MCS	−0.35**	−0.31**	−0.11	−0.41**
SF12-PCS	−0.32**	− 0.28**	− 0.04	− 0.37**
Age	− 0.11	− 0.13	− 0.05	−0.15*

*p < 0.05; **p < 0.001

*MAX-PC* Memorial anxiety scale for prostate cancer, *PCA* Prostate cancer anxiety subscale, *PSAA* PSA anxiety subscale, *FOR* Fear of recurrence subscale

*HADS* Hospital Anxiety and Depression scale, *HADS-Anxiety* HADS anxiety subscale, *HADS-Depression* HADS Depression subscale

*SF12* Short Form-12, *SF12-PCS* SF12 physical component subscale, *SF12-MCS* SF12 mental component subscale

## Discussion

Originally developed and validated in English, the MAX-PC is a useful PROM for evaluating prostate-cancer related anxiety [17]. To our knowledge, this scale has not been translated to French or validated in a sample of French prostate cancer patients. Moreover, only a limited number of studies have assessed the psychometric properties of the MAX-PC [12, 17, 35–37]. In our study, we investigated the three-factor structure defined in the original English validation study by Roth et al [17]. Our results show that the French version of the MAX-PC has adequate reliability, validity and internal structure.

As shown in Table 2, item 2 (“Even though it’s a good idea, I found that getting a PSA test scared me”) loaded higher on the PSAA subscale than on the PCA subscale (0.42 vs 0.45) in the EFA, most likely because this item refers specifically to PSA testing. Nevertheless, we decided to retain the three-factor structure defined in the original validation study, because in the CFA this factor structure had better fit than the modified factor structure in which item 2 was moved from the PCA subscale to the PSAA subscale and also than the structure with item 2 cross-loaded by two factors (PCA and PSAA). The results of the MI showed redundant elements in the model. The highest MI values were between items 2 and 4 (14.2) and items 13 and 14 (10.1). We chose to estimate their residual covariances as free parameters, which improved the fit indices of the original structure (RMSEA = 0.073 (90% Confidence Interval: 0.043–0.098), CFI = 0.964, and TLI = 0.958).

The MAX-PC and two of its subscales (PCA and FOR) were found to have adequate reliability. Cronbach’s alpha coefficients were higher in our validation study than in the original English validation study [17], the Dutch validation study [36], and the Italian validation study [37]. Although Cronbach’s alpha coefficient for the PSAA subscale was low (< 0.70), it was higher in our validation study than in the three aforementioned validation studies. Only the Chinese version of the MAX-PC was found to have a higher reliability than our French version, this being especially true for the PSAA subscale (α = 0.82) [12]. In our sample, the poor performance of the PSAA subscale was likely due to strong floor effects (as 69.2% of respondents had the lowest possible score) and to the fact that this subscale contains only three items. In the Italian validation study, the three items of the PSAA subscale were deleted from the questionnaire on the grounds that they were not relevant to patients on active surveillance [37]. However, two other validation studies found good psychometric properties of the PSAA subscale. In the study by Nelson et al. [35], which focused on 101 African-American patients, the MAX-PC had a Cronbach’s alpha coefficient of 0.71, and the MAX-PC total score was significantly correlated with the HADS-Anxiety subscale score (r = 0.35; *p* < 0.01). The authors explained these results by the fact that African-American men have greater distrust in medical institutions, which often leads them to delay or to repeat their PSA test [35]. In the Chinese validation study, Huang et al. also explained the good psychometric properties of the PSAA subscale by the fact that Chinese patients tend to distrust their doctors [12]. For our part, we decided not to delete the three items of the PSAA subscale because we had no information on the stage of the disease, the treatment administered to the patient, and, most importantly, the date of the last PSA test. In fact, we considered the possibility that some of the respondents were not anxious about taking the next PSA test because they had been diagnosed fairly recently (PSA-related anxiety can develop long after diagnosis) or because they had just taken the test. We also took into account the fact that French patients generally trust their doctors [38], and are therefore less likely to delay or repeat the PSA test.

The MAX-PC total score, the PCA subscale score, and the FOR subscale score were found to have adequate construct validity. Moreover, the positive correlation of the MAX-PC total score with the HADS total score, the HADS-Anxiety subscale score, and the HADS-Depression subscale score indicated good convergent validity. As expected, the negative correlation of the MAX-PC total score with the SF12-MCS subscale score was significant. The latter finding is concordant with the Dutch validation study, which is the only other MAX-PC validation study to have included the SF12 in the questionnaire [36].

Our study has some limitations that must be acknowledged. First, test-retest reliability could not be assessed due to the cross-sectional design of the study. Second, no personal data (except for age) or medical data (such as cancer stage, PSA outcome, or treatment history) were collected from our participants, making it impossible to define anxiety profiles based on personal and medical parameters. Nevertheless, the two independent subsamples of prostate cancer patients were relatively similar (participants were similar in age and were all recruited within 12 months after diagnosis). Another limitation of our study is that participants were recruited through advocacy organizations, suggesting that our sample may not be representative of the population of prostate cancer patients. It should be noted that the scale showed acceptable fit even though our sample had less than 100 patients [39]. The goodness of fit indices would likely have been better if the CFA had been performed on a larger sample. Nevertheless, ours is the first study to validate a French version of this prostate cancer-specific anxiety scale.

## Conclusion

Our study suggests that the MAX-PC is a reliable PROM for evaluating prostate cancer-related anxiety in French men. In the future, the properties of the MAX-PC should be tested in longitudinal studies. Moreover, the scale should be made available in the clinical context to help practitioners understand the determinants of prostate cancer-related anxiety, after confirming its psychometric properties on a larger sample with more sociodemographic and clinical information.

## Data Availability

The datasets generated during and/or analysed during the current study are available from the corresponding author on reasonable request.

## Abbreviations

ANAMACaP: Association Nationale des MAlades du Cancer de la Prostate
BSI: Brief Symptom Inventory
CFA: Confirmatory Factory Analysis
CFI: Comparative Fit Index
CNIL: Comission Nationale de l’Information et des Libertés
DT: Distress Thermometer
EFA: Exploratory Factor Analysis
FOR: Fear Of Recurrence
HADS: Hospital Anxiety and Depression Scale
KMO: Kaiser-Meyer-Oklin
MAX-PC: Memorial Anxiety Scale for Prostate Cancer
MCS: Mental Component Score
MI: Modification indices
PCA: Prostate Cancer Anxiety
PCS: Physical Component Score
PROMs: Patient Reported Outcome Measures
PSA: Prostate-Specific Antigen
PSAA: Prostate-Specific Antigen Anxiety
RMSEA: Root Mean Square Error of Approximation
SF12: Short Form-12
TLI: Tucker-Lewis Index
